# Genetic Variations in miR-30 Family Member Regulatory Regions Are Associated with Breast Cancer Risk in a Chinese Population

**DOI:** 10.1155/2020/8781348

**Published:** 2020-03-27

**Authors:** Jing Zhou, Lijuan Wang, Sijun Liu, Wen Zhou, Yue Jiang, Jiangbo Du, Juncheng Dai, Guangfu Jin, Hongxia Ma, Zhibin Hu, Jiaping Chen, Hongbing Shen

**Affiliations:** ^1^Department of Epidemiology, International Joint Research Center on Environment and Human Health, Center for Global Health, School of Public Health, Nanjing Medical University, Nanjing, China; ^2^Statistical Room, Medical Quality Control Department, Northern Jiangsu People's Hospital and Clinical Medical College of Yangzhou University, Yangzhou, China; ^3^Jiangsu Key Lab of Cancer Biomarkers, Prevention and Treatment, Collaborative Innovation Center for Cancer Medicine, Nanjing Medical University, Nanjing, China; ^4^Department of Social Medicine and Health Education, School of Public Health, Nanjing Medical University, Nanjing, China; ^5^State Key Laboratory of Reproductive Medicine, Nanjing Medical University, Nanjing, China

## Abstract

MicroRNAs (miRNAs) of the miR-30 family are closely linked with tumor metastasis and play key roles in the complex malignant phenotypes of cancers by targeting many tumor-related genes. Deregulated expression of miR-30 family members has been commonly observed in breast cancer. However, associations between the genetic variants in the regulatory region of miR-30 family and the risk of breast cancer are still limited, especially in the Chinese Han population. In the present study, we conducted a case-control analysis wherein 1064 breast cancer patients and 1073 healthy controls underwent genotyping of 10 SNPs in the regulatory region of miR-30 family members. Multivariate logistic regression analyses illustrated that the rs763354 variant in the miR-30a regulatory region was linked with a significant decrease in breast cancer risk in an additive model (adjusted OR = 0.86, 95% CI: 0.75-0.98, *P* = 0.022). Further, eQTL analyses also indicated that this SNP was associated with miR-30a expression levels in breast cancer samples compiled in the TCGA database (*P* = 0.020). The Kaplan-Meier plotter showed that breast cancer patients with higher miR-30a expression have significantly better outcomes than do patients expressing low levels of this miRNA (HR = 0.75, 95% CI: 0.61-0.91, *P* = 0.0041). Together, these findings suggest that the miR-30a rs763354 SNP is an important regulator of breast cancer risk, thus making it a potentially viable prognostic biomarker and one that can be used to guide therapeutic treatment in affected patients.

## 1. Introduction

Breast cancer remains the leading cause of cancer morbidity and mortality among women, with an estimated 2.1 million newly diagnosed cases globally in 2018 alone [1]. In China, the incidence of female breast cancer is continuously increasing in recent years, with an estimated accounting for 15% of newly diagnosed cancers among women [2]. While earlier detection efforts and the more widespread application of comprehensive therapeutic regimens have led to significant reductions in breast cancer mortality in recent years, the precise mechanisms governing the development and progression of this disease remain incompletely understood.

Recently, accumulating evidence indicates that miRNAs may act as either tumor suppressors or oncogenes in the genesis of diverse cancer types [3, 4]. The dysregulated expression of certain miRNAs, including miR-30 family members, has been closely linked to the onset and progression of breast cancer [5–7]. The miR-30 family includes 6 mature miRNAs (miR-30a, miR-30b, miR-30c-1, miR-30c-2, miR-30d, and miR-30e) that are separately encoded on chromosomes 1, 6, and 8. In most studies, these miR-30 family members have been shown to function as tumor suppressors that act to constrain the development of various cancers [7–15]. Among the miR-30 members, miR-30a had drawn much attention due to its functions in inhibiting primary tumor growth or metastasis formation in several cancer types, especially in breast cancer [8–12]. When miR-30a expression was decreased in breast tumors, this was linked with higher rates of lymph node metastasis, p53 inactivation, and poorer patient prognosis [6]. In other studies, miR-30b was shown to suppress CCNE2 expression in HER2-positive breast cancer cells, thereby suppressing their growth and inducing G1 phase cell cycle arrest [13]. Still, other work has found that miR-30c can inhibit the induction of Serpine 1 by TGF-*β*-induced Serpine 1, driving fibrin degradation and disrupting angiogenesis within the tumor microenvironment in order to constrain tumor progression [14]. The migration and invasion of the BT474 and MDA-MB-231 cancer cell lines were shown to be impaired by miR-30d, which was able to target KLF11 and the STAT3 pathway in order to regulate tumor cell epithelial-mesenchymal transition [7]. IRS1 has also been shown to be targeted by miR-30e, leading to impaired breast cancer tumor growth and chemoresistance [15].

In contrast to these tumor suppressor roles, certain studies have identified context-dependent roles for miR-30 family members as oncogenic factors that promote tumor development [16–18]. For example, when miR-30a was overexpressed in ovarian cancer cells, this was associated with impaired FOXL2 expression and enhanced BCL2A1, IER3, and cyclin D2 expressions, thereby driving tumorigenesis [16]. In addition, the ability of miR-30c to target NOV/CCN3 has been linked with metastatic breast cancer cell invasive activity [17]. Further studies have also shown that miR-30b and miR-30d are capable of driving melanoma cell metastasis via suppressing GALNT7, leading to enhanced production of IL-10, which can in turn impair local immune cell activity and recruitment [18].

While the exact mechanisms that give rise to miRNA dysregulation in the context of cancer remain poorly understood, genetic variants in the regulatory regions for specific miRNAs have been linked with the miRNA transcription and with consequent changes in cancer risk. For example, the rs12740674 variant in the enhancer region of miR-1262 may reduce the miR-1262 expression in lung tissue through chromosomal looping, and this in turn has been associated with elevated lung cancer risk [19]. Similarly, the CC genotype in the rs10877887 position of the let-7 miRNA has been shown to be closely associated with cervical cancer risk and to decreased let-7 expression, with this variant being associated with reduced binding of the transcription factor MZF1 to the let-7 regulatory region, thereby reducing the expression of this miRNA [20]. On account of the essential roles of miR-30 family members in the genesis or development of diverse cancers, SNPs in the miR-30 are prime target as candidate markers that affect tumor susceptibility. Therefore, in the present study, we aimed to detect SNPs in the regulatory regions controlling miR-30 family member expression that are linked with breast cancer risk in women. Specifically, we focused on 10 SNPs in these regions, comparing the relative frequencies of these SNPs in breast cancer patients from a Chinese Han population with healthy controls through a case-control approach. We hope that the results will offer valuable insight into the mechanisms regulating breast cancer development, guiding future efforts to prevent or treat this disease in an individualized manner.

## 2. Methods

### 2.1. Study Population

This case-control study was approved by the Review Board of Nanjing Medical University. All the procedures were carried out according to the predefined guidelines and ethical criteria. In total, this study included 1064 patients with breast cancer and 1073 healthy controls, as described previously [21]. All study participants were females of Han Chinese ethnicity and provided informed consent to participate in the present study. A structured questionnaire was used to obtain information from all participants regarding their demographic information, reproductive and menstrual history, and history of environmental exposures. For patients with breast cancer, the information regarding progesterone and estrogen receptor (PR and ER) status was collected from their medical records.

### 2.2. SNP Selection and Genotyping

The miR-30 family contains 5 members and 6 mature miRNAs (miR-30a, miR-30b, miR-30c-1, miR-30c-2, miR-30d, and miR-30e). We utilized the International HapMap Project (http://www.hapmap.org), dbSNP (http://www.ncbi.nlm.nih.gov/-projects/SNP/), and UCSC (http://genome.ucsc.edu/) databases in order to identify SNPs that were located within a 10 kb distance upstream of these pre-miRNA chromosomal regions. Candidate SNPs were then identified through the identification of those SNPs, which had, in a Han Chinese population, appropriate linkage disequilibrium (LD) and MAF values (*r*^2^ < 0.8 and MAF ≥ 0.05, respectively). Using these criteria, we were able to identify 10 distinct SNPs that were upstream of these pre-miR-30 family members (rs763354, rs852963, rs852964, rs928508, rs12743517, rs3767950, rs12208417, rs16881192, rs17709260, and rs7846345).

We then used the Illumina Infinium BeadChip platform (Illumina) in order to genotype all 2137 study participants for the presence of these 10 SNPs, which were successfully detected with >95% call rates in all cases and controls. The genotyping was conducted in a manner that blinded the case/control status of a given participant sample, with approximately equivalent numbers of the breast cancer patient and control participant samples being tested per assay. In addition, two blank controls were included in each assay.

### 2.3. Statistical Analyses

Student's *t*-tests and *χ*^2^ tests were used to assess differences between case and control groups with respect to continuous and categorical demographic variables, respectively. For each SNP of interest, the Hardy-Weinberg equilibrium (HWE) for this genetic variant was analyzed in controls based upon a goodness-of-fit *χ*^2^ test for each SNP. The relationship between a given SNP and breast cancer was then quantified through logistic regression analysis that calculated the odds ratios (ORs) and 95% confidence intervals (CIs) for this relationship after correcting for age, age at menarche, and menopausal status. A *Q* test was used to assess heterogeneity among subgroups through a stratified analytical approach, while Pearson correlation test and Turkey HSD multicomparison method were used to assess the relationship between miRNA expression and breast cancer sample genotype in the TCGA database. All statistical analyses were performed using R software (version 3.2.2; The R Foundation for Statistical Computing). Statistical tests were two-sided, with *P* < 0.05 as the significance threshold.

### 2.4. Bioinformatics Analysis

The association between candidate SNPs and miRNA regulatory elements was assessed by identifying potential regulatory elements and functional variants with HaploReg v4.1 (http://archive.broadinstitute.org/mammals/haploreg/haploreg.php). TCGA datasets were subjected to expression quantitative trait loci (eQTL) analyses in order to examine the relationship between a given genotype and associated phenotypic changes. Level 1 genotypic data (Affymetrix 6.0 array) from 1011 breast cancer samples was imputed using IMPUTE2, while corresponding level 3 miRNA expression data from 669 breast cancer samples and 86 paracancerous control tissue samples were also obtained from TCGA. The EdgeR package was used to normalize these data after which miR-30 family member expression data were subjected to a log2 transformation and additional analysis.

### 2.5. Online Kaplan-Meier Plotter

The prognostic relevance of miR-30 family member expression in breast cancer patients was examined using the Kaplan-Meier plotter (http://kmplot.com/analysis/). Briefly, patient miRNA expression and overall survival data were obtained from public datasets after which patients were separated into two groups based upon the level of expression for a given miRNA in these patient samples. Kaplan-Meier survival curves were then used to assess the prognostic relationship between miRNA expression and patient survival, with HRs, corresponding 95% CIs, and log-rank *P* values being calculated as appropriate [22].

## 3. Results

### 3.1. The Relationship between Selected SNPs and Breast Cancer Risk

The demographic information for breast cancer cases and controls (*n* = 1064 and *n* = 1073, respectively) are compiled in Supplementary [Supplementary-material supplementary-material-1]. No significant differences in age were evident between these breast cancer cases and controls (*P* > 0.05), but patients with breast cancer had significantly earlier menarche and significantly later first live births relative to controls (*P* < 0.0001).

The chromosomal locations and disease associations of the 10 miR-30 family member SNPs are given in Table 1. All genotype distributions in the controls were consistent with expected frequencies based upon the Hardy-Weinberg equilibrium model (*P* > 0.05). Of these 10 SNPs, we determined that the rs763354 SNP within the miR-30a regulatory region was associated with a significant reduction in breast cancer risk in an additive model (rs763354 adjusted OR = 0.86, 95% CI: 0.75-0.98, *P* = 0.022). None of the other 9 analyzed SNPs exhibited any significant association with breast cancer risk (Table 1).

The relationship between the rs763354 SNP and risk of breast cancer was additionally analyzed based upon dominant, codominant, and recessive models (Table 2). This analysis revealed that under both codominant and recessive models, this SNP was associated with a significant reduction in breast cancer risk (GG : AA, OR = 0.72, 95% CI: 0.55-0.95, *P* = 0.020; recessive model OR = 0.77, 95% CI: 0.60-0.99, *P* = 0.042).

We additionally examined the relationship between the rs763354 SNP and risk of breast cancer according to age, age at menarche, age at first live birth, and menopausal status in a stratified analysis. As shown in Table 3, the breast cancer risk associated with variant AG/AA genotypes (vs. the GG genotype) was significantly lower among women with later menopause (adjusted OR = 0.75; 95% CI: 0.58–0.97), ER-negative women (adjusted OR = 0.73; 95% CI: 0.61–0.87), and PR-negative women (adjusted OR = 0.74; 95% CI: 0.62–0.89). However, paired comparisons did not reveal any significant heterogeneity (*P* > 0.05).

### 3.2. In Silico Analysis

Using HaploReg v4.1, we next conducted annotation of the rs763354 SNP, which is found 2367 bp upstream of miR-30a in the LINC00472 gene. The rs763354 variant allele is associated with alterations in the SOX and AIRE regulatory binding motif in this region (Supporting Information [Supplementary-material supplementary-material-1]). In addition, histone modification peaks indicated that rs763354 was located in an enhancer element based upon the presence of H3K4me1 and H3K4me3 histone modifications in this region in breast myoepithelial primary cells and breast variant human mammary epithelial cells (vHMECs).

**Figure 1:**
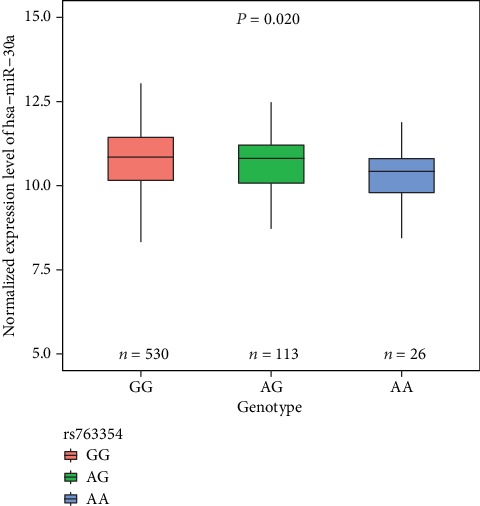
eQTL analysis of rs763354 in the TCGA breast cancer dataset. The boxplot showing the effects of the genotypes of rs763354 on miR-30a expression levels by using Pearson correlation test in 669 TCGA breast cancer tumor tissues (*P* = 0.020).

The relevance of this rs763354 SNP to miR-30a expression in breast cancer was further assessed through genotypic and phenotypic analyses of 669 breast cancer tissue samples from TCGA breast cancer (BRCA) datasets. The expression quantitative trait locus (e-QTL) analysis was performed using a Pearson correlation test of rs763354 genotype with log-transformed expression level of hsa-mir-30a. This analysis revealed a significant association between rs763354 genotype and the expression of miR-30a, with the rs763354-A allele being associated with reduced hsa-miR-30a expression (*P* = 0.020, Figure 1). Meanwhile, we used Turkey HSD multicomparison method to compare the expression difference of hsa-miR-30a between groups, and we found that the difference between rs763354-GG group and rs763354-AA group was statistically significant (*P* = 0.04), while no differences were detected between GG vs AG (*P* = 0.60) or AG vs AA (*P* = 0.18) groups.

In short, the results indicated that the rs763354 was probably involved in the regulation of miR-30a expression.

### 3.3. The Relationship between miR-30a Expression and Breast Cancer Patient Outcomes

**Figure 2:**
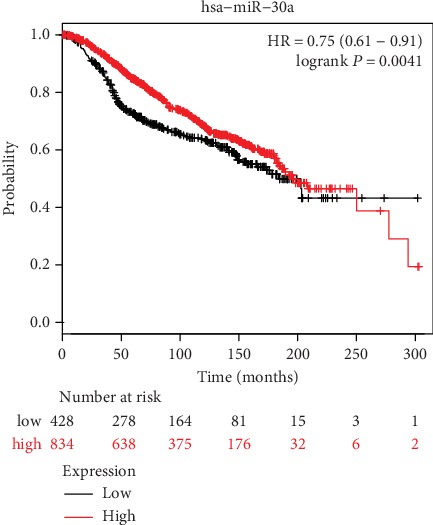
Kaplan-Meier survival curve of breast cancer patients according to the expression of miR-30a by using online Kaplan-Meier plotter. A Kaplan-Meier survival analysis reveals that breast cancer patients with higher miR-30a expression have significantly better outcomes than those with low expression (*P* = 0.0041).

The association between the miR-30a expression and prognosis of breast cancer was assessed by using the Kaplan-Meier plotter (http://kmplot.com/analysis/), which combined the follow-up information and expression profiles [22]. Patients were split into high expression and low expression group according to the median of miR-30a expression. A subsequent log-rank analysis of the resultant Kaplan-Meier curve revealed that higher miR-30a expression was associated with significantly better breast cancer patient outcomes than low miR-30a expression (HR = 0.75, 95% CI: 0.61-0.91, *P* = 0.0041, Figure 2). In addition, the miR-30a expression was also reduced between 86 paired breast cancer tissues and normal paracancerous tissues in the TCGA breast cancer (BRCA) datasets (*P* = 0.003, Supporting Information Figure 1). These findings indicate that miR-30a may function as a tumor suppressor in breast cancer.

## 4. Discussion

In the present report, we employed a case-control study in order to examine the relationship between 10 different SNPs present in the regulatory regions of miR-30 family member genes and breast cancer risk. We found that the rs763354 SNP in the miR-30a regulatory region may be a candidate variant associated with breast cancer susceptibility in a Chinese Han population. Moreover, we found that the SNP rs763354 was correlated with the expression of miR-30a in an eQTL analysis based upon TCGA breast cancer datasets. Lastly, we found that elevated miR-30a expression may be associated with better breast cancer patient outcomes through a Kaplan-Meier plotter-based analysis.

The rs763354 is 2367 bp upstream of pre-miR-30a. Previous studies have defined miR-30a as a putative tumor suppressor that is frequently dysregulated in cancers including osteosarcoma and cancers of the breast, lung, and head and neck [11, 23–28]. One mechanism whereby miR-30 may modulate cancer development is via inhibiting cell proliferation and invasion and by inducing apoptotic cell death [24–27]. Indeed, miR-30a overexpression is associated with reduced BCL-2 expression in non-small-cell lung cancer, leading to the increased apoptotic death of these cells [24]. This miRNA is also associated with reductions in the expression of RPA1 and associated enhancement of p53 expression, leading to inhibition of DNA replication and apoptotic death in cancer cells [25]. Further work also suggests that miR-30a can target FOXD1 in human osteosarcoma cells so as to inhibit their proliferation [26]. Furthermore, in xenograft models of head and neck cancer, miR-30a has been shown to suppress growth receptor expression and to slow tumor growth [27]. Expression of miR-30a has been shown to be markedly reduced in patients suffering from triple-negative breast cancer with mutations in the *TP53* gene, and this expression was negatively correlated with patient survival. Inactivation of p53 in these patients was associated with reduced miR-30a expression and an associated decrease in miR-30a-mediated suppression of ZEB2 expression, thereby leading to enhanced breast cancer cell plasticity, migration, and metastasis [6]. Other targets of miR-30a in breast cancer have also been identified as potential links between this miRNA and the regulation of metastatic progression, including vimentin, MTDH, and Eya2 [11, 23, 28]. In the present report, we used the Kaplan-Meier plotter database in order to examine the relationship between miR-30a expression and breast cancer patient survival, revealing that elevated expression of this miRNA is associated with improved overall survival. The expression of miR-30a was also reduced in tumor tissue samples relative to normal paracancerous tissue samples in the TCGA database. These results thus suggest that miR-30a may function as a tumor suppressor in breast cancer.

The rs763354 SNP exhibits a strong linkage with the rs2222722 SNP in the miR-30a promoter region (*r*^2^ = 0.89, D′ = 0.99), and this latter SNP has previously been shown to be associated with colorectal cancer risk in an Iranian population and with nephrotic syndrome risk in a Chinese cohort [29, 30]. The rs763354 SNP has also previously been shown to be linked with non-small-cell lung cancer risk in a Chinese population [31]. In the present report, we found this SNP to be associated with a reduction in breast cancer risk, with these risk effects remaining significant even in particular patient subgroups including women who were ER-negative, PR-negative, or who had reached menopause at a later age. No significant heterogeneity was detected for these paired comparisons, suggesting that these variables were thus unrelated to the risk effect for the rs763354 SNP.

According to the HaploReg database, the rs763354 SNP is in an enhancer region, and the genotypic variation at this site is associated with altered AIRE and SOX regulatory motif binding. SOX2 is known to be a key regulator of a wide range of different miRNAs, thereby regulating many critical genes involved in the epithelial-to-mesenchymal transition (EMT) [32]. AIRE has also been found to be a key regulator of miRNA expression in medullary thymic epithelial cells [33]. An analysis of histone modifications present at rs763354 in breast vHMECs and breast myoepithelial primary cells suggested that this region bears H3K4me1 and H3K4me3 histone marks consistent with a role as an enhancer element. Further, eQTL analyses indicated that an A allele at the rs763354 site was associated with decreased expression of miR-30a in a cohort of 669 breast cancer tumor samples. Together, these results thus suggested that the rs763354 SNP may regulate miR-30a expression via controlling transcription factor binding to this region, although further functional assays will be needed to confirm this result.

This study has some limitations. First, we conducted only one stage case–control study with 1064 breast cancer cases and 1073 healthy controls. Studies with large sample size and more diverse different populations are needed to validate the current results. Second, our results provide bioinformatics-based evidence, but the biological effect of the rs763354 variant on miR-30a expression and function was not investigated in this study and required further clarification.

In conclusion, in this study, we assessed the relationship between 10 different SNPs present in genomic regions associated with the regulation of miR-30 family member gene expression and breast cancer risk in a Chinese Han population. Our results revealed that the rs763354 SNP in the miR-30a regulatory region may be a candidate variant associated with breast cancer risk in this population. However, further studies in larger patient cohorts with different ethnic backgrounds and additional functional validation efforts will be needed to fully confirm these findings.

## Figures and Tables

**Table 1:** Associations between ten SNPs in the miR-30 family members and breast cancer risk.

SNP	Chr	Position (hg38)	Location	Alleles^a^	Cases^b^ (*n* = 1064)	Controls^b^ (*n* = 1073)	Call rate (%)	MAF^c^ (case/control)	HWE^d^	OR (95% CI)^e^	*P* value^e^
rs763354	6	71405918	2.4 kb upstream of pre-miR-30a	G/A	155/478/407	188/499/365	97.89	0.38/0.42	0.447	0.86 (0.75-0.98)	0.022
rs852963	6	71412803	9.3 kb upstream of pre-miR-30a	G/A	69/341/652	45/353/672	99.77	0.23/0.21	0.926	1.09 (0.93-1.26)	0.293
rs852964	6	71413926	10.4 kb upstream of pre-miR-30a	G/A	150/482/431	127/489/457	99.95	0.37/0.35	0.893	1.12 (0.98-1.28)	0.085
rs928508	1	40757742	458 bp upstream of pre-miR-30c-1	A/G	239/515/308	242/512/319	99.91	0.47/0.46	0.197	1.05 (0.92-1.19)	0.462
rs12743517	1	40759682	2.4 kb upstream of pre-miR-30c-1	C/A	201/516/346	189/519/365	99.95	0.43/0.42	0.851	1.03 (0.91-1.17)	0.647
rs3767950	1	40767541	10.3 kb upstream of pre-miR-30c-1	C/A	160/472/430	158/481/432	99.81	0.37/0.37	0.214	1.02 (0.9-1.16)	0.758
rs12208417	6	71385948	9.0 kb upstream of pre-miR-30c-2	C/A	241/527/295	233/549/290	99.91	0.47/0.47	0.392	0.99 (0.87-1.12)	0.832
rs16881192	6	71386063	9.1 kb upstream of pre-miR-30c-2	A/C	83/420/553	80/400/586	99.30	0.28/0.26	0.305	1.1 (0.95-1.27)	0.197
rs17709260	8	134810212	5.3 kb upstream of pre-miR-30d	A/G	12/144/908	3/134/936	100	0.08/0.07	0.616	1.23 (0.96-1.57)	0.107
rs7846345	8	134814337	9.4 kb upstream of pre-miR-30d	G/C	180/526/358	185/505/383	100	0.42/0.41	0.411	1.01 (0.89-1.15)	0.854

^a^Major/minor allele. ^b^Major homozygote/heterozygote/rare homozygote between cases and controls. ^c^Minor allele frequency (MAF). ^d^*P* values for the Hardy-Weinberg equilibrium (HWE) test. ^e^Logistic regression analysis with adjustment for age, age at menarche, and menopausal status in the additive model. Chr: chromosome; OR: odds ratio; CI: confidence interval.

**Table 2:** Associations between rs763354 and breast cancer risk in three different genetic models.

SNP	Genetic models	Genotypes	Cases	Controls	OR (95% CI)^a^	*P* value^a^
rs763354	Codominnant	GG	407	365	1.00	
		AG	478	499	0.89 (0.73-1.09)	0.253
		AA	155	188	0.72 (0.55-0.95)	0.020
	Dominant	GG	407	365	1.00	
		AG/AA	633	687	0.84 (0.70-1.02)	0.077
	Recessive	GG/AG	885	864	1.00	
		AA	155	188	0.77 (0.60-0.99)	0.042

^a^Logistic regression analysis with adjustment for age, age at menarche, and menopausal status in the codominant model, dominant model, and recessive model, respectively.

**Table 3:** Stratification analysis of association between rs763354 in the regulatory region of miR-30a and risk of breast cancer.

Characteristics	Case	Control	OR (95% CI)^a^	*P*	*P*_het_^b^
Age					0.226
≤51	232/276/97	198/253/100	0.92 (0.78-1.09)	0.344	
>51	175/202/58	167/246/88	0.79 (0.65-0.95)	0.015	
Age at menarche					0.869
≤16	298/356/116	222/300/115	0.87 (0.74-1.01)	0.063	
>16	103/112/37	143/198/72	0.85 (0.67-1.06)	0.151	
Age at first live birth					0.652
≤24	134/175/53	176/247/91	0.89 (0.73-1.09)	0.258	
>24	246/274/93	176/242/90	0.84 (0.70-1.00)	0.052	
Age at natural menopause					0.435
≤49	72/75/23	81/112/32	0.88 (0.65-1.19)	0.411	
>49	102/126/37	84/136/54	0.75 (0.58-0.97)	0.030	
Menopausal status					0.938
Premenopausal	198/231/76	181/229/89	0.87 (0.72-1.04)	0.117	
Postmenopausal	204/243/76	180/260/93	0.86 (0.72-1.02)	0.091	
ER status					0.107
Positive	179/224/78		0.89 (0.76-1.04)	0.152	
Negative	163/158/45		0.73 (0.61-0.87)	0.0005	
PR status					0.159
Positive	192/225/81		0.88 (0.75-1.03)	0.126	
Negative	149/156/44		0.74 (0.62-0.89)	0.001	

^a^Per allele odds ratio (OR) and 95% confidence interval (CI) adjusted for age, age at menarche, and menopausal status where appropriate. ^b^*P* for heterogeneity test.

## Data Availability

The data used to support the findings of this study are available from the corresponding author upon request.

## Supplementary Materials

Supplementary Materials Supplementary Table 1: demographic and selected variables in breast cancer and control patients. Supplementary Table 2: summary of the functional analyses for the linkage disequilibrium (LD) block containing rs763354 (*r*^2^ = 1). Supplementary Figure 1: the expression of miR-30a between 86 paired tumor and nontumor tissues in the TCGA breast cancer (BRCA) datasets.
