# Supplementary material for: Genetic Variations in miR-30 Family Member Regulatory Regions Are Associated with Breast Cancer Risk in a Chinese Population
Source: Biomed Res Int. 2020 Mar 27;2020:8781348. doi: 10.1155/2020/8781348 (PMC7140140; doi:10.1155/2020/8781348)

Supplementary Table 1. Demographic and selected variables in breast cancer and control patients

**Variables**	**Cases**[a] **(N=1064)**	**Controls**[b] **(N=1073)**	P^c
Age, year (mean ± SD)	50.89±11.44	51.51±11.82	0.217
Age at menarche, year (mean ± SD)	15.23±1.93	16.11±1.94	<0.0001
Age at first live birth, year (mean ± SD)	25.57±3.25	24.62±3.35	<0.0001
Age at natural menopause, year (mean ± SD)	49.72±3.53	49.59±3.98	0.606
Menopausal status			<0.0001
Premenopausal	523(49.71)	525(49.86)	
Natural menopause	451(42.87)	505(47.96)	
Unnatural menopause	78(7.41)	23(2.18)	
Estrogen receptor (ER)[d]			
Positive	490(56.39)		
Negative	379(43.61)		
Progesterone receptor (PR) [d]			
Positive	506(58.23)		
Negative	363(41.77)		

[a] Cases were consecutively recruited from the First Affiliated Hospital of Nanjing Medical University, the Cancer Hospital of Jiangsu Province and the Gulou Hospital, Nanjing, China, from Jan 2004 to April 2010;

[b] Controls were randomly selected from a cohort of more than 30,000 participants in a community-based screening program for non-infectious diseases conducted in Jiangsu Province;

[c] T-tests and $\chi 2$ tests were used for continuous or categorical variables, respectively;

[d] ER and PR status information was available in 869 breast cancer cases.

Supplementary Table 2. Summary of the functional analyses for the linkage disequilibrium (LD) block containing rs763354 (r^2= 1)

chr	pos (hg38)	LD (r^2)	variant	Ref	Alt	ASN freq	Promoter histone marks	Enhancer histone marks	DNAse	Motifs changed	GRASP QTL hits	Selected eQTL hits
6	71405918	1	rs763354	C	T	0.39		12 tissues		AIRE,Sox	1 hit	1 hit
6	71409013	1	rs7349905	G	C	0.39		14 tissues	SKIN	AP-1,Mxi1,SREBP		
6	71411759	1	rs9342836	T	C	0.39	KID, GI, LNG	11 tissues		11 altered motifs		1 hit
6	71414369	1	rs1918478	G	T	0.39	LIV, KID, LNG	9 tissues		GATA,LUN-1		

LD, r^2 between the variant and required one; Ref, reference allele; Alt, alternative allele; ASN, populations from Asia in 1000 genomes project; Promoter histone marks, Cell types where SNPs are in histone-marks of promoter; Enhancer histone marks, cell types where SNPs are in histone-marks of enhancer; DNase, cell types where SNPs are in the accessible region of DNase.

Supplementary Figure 1: The expression of miR-30a between 86 paired tumor and non-tumor tissues in the TCGA breast cancer (BRCA) datasets

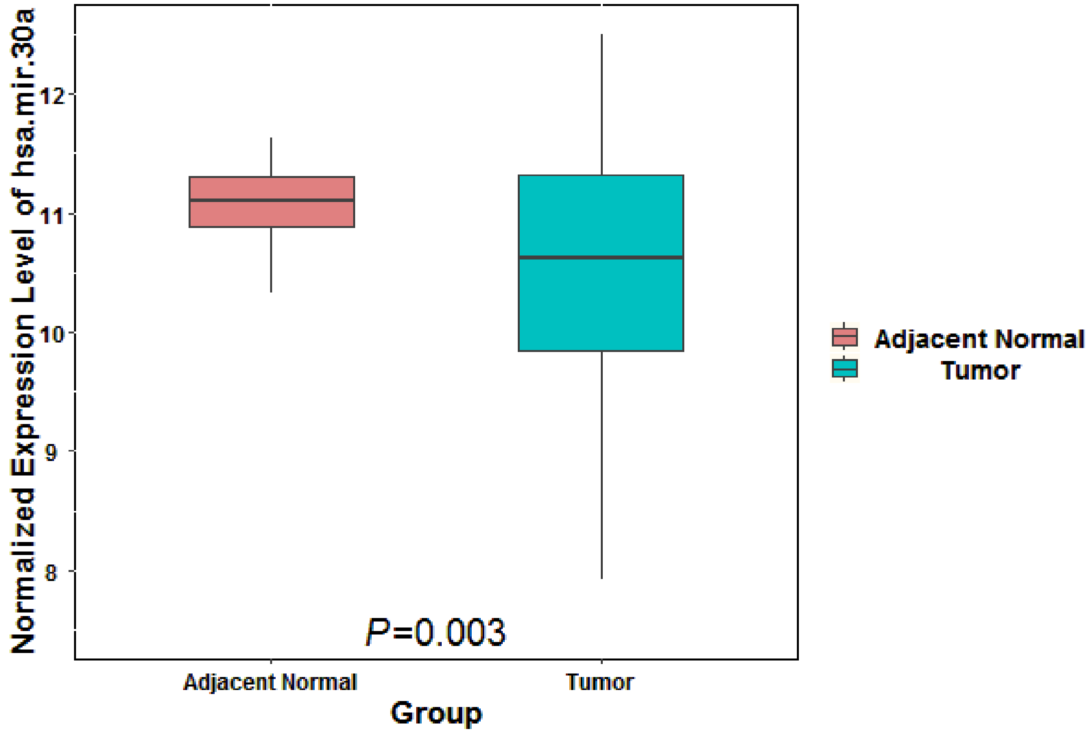

Supplement: Supplementary Materials — Supplementary Table 1: demographic and selected variables in breast cancer and control patients. Supplementary Table 2: summary of the functional analyses for the linkage disequilibrium (LD) block containing rs763354 (r2 = 1). Supplementary Figure 1: the expression of miR-30a between 86 paired tumor and nontumor tissues in the TCGA breast cancer (BRCA) datasets. [file 8781348.f1.pdf]
